# Children with burn injuries-assessment of trauma, neglect, violence and abuse

**DOI:** 10.5249/jivr.v3i2.91

**Published:** 2011-07

**Authors:** Michael H. Toon, Dirk M. Maybauer, Lisa L. Arceneaux, John F. Fraser, Walter Meyer, Antoinette Runge, Marc O. Maybauer

**Affiliations:** ^a^Critical Care Research Group and The University of Queensland, The Prince Charles Hospital at Brisbane, Australia.; ^*b*^Department of Anesthesia and Intensive Care, Philipps University of Marburg, Marburg, Germany.; ^*c*^Department of Anesthesiology, The University of Texas Medical Branch and Shriners Burns Hospital at Galveston, USA.; ^*d*^Department of Surgery, The University of Texas Medical Branch and Shriners Burns Hospital at Galveston, USA.; ^*e*^Department of Psychiatry, The University of Texas Medical Branch and Shriners Burns Hospital at Galveston, USA.

## Abstract

Burns are an important cause of injury to young children, being the third most frequent cause of injury resulting in death behind motor vehicle accidents and drowning. Burn injuries account for the greatest length of stay of all hospital admissions for injuries and costs associated with care are substantial.

The majority of burn injuries in children are scald injuries resulting from hot liquids, occurring most commonly in children aged 0-4 years. Other types of burns include electrical, chemical and intentional injury. Mechanisms of injury are often unique to children and involve exploratory behavior without the requisite comprehension of the dangers in their environment.

Assessment of the burnt child includes airway, breathing and circulation stabilization, followed by assessment of the extent of the burn and head to toe examination. The standard rule of 9s for estimating total body surface area (TBSA) of the burn is inaccurate for the pediatric population and modifications include utilizing the Lund and Browder chart, or the child's palm to represent 1% TBSA. Further monitoring may include cardiac assessment, indwelling catheter insertion and evaluation of inhalation injury with or without intubation depending on the context of the injury. Risk factors and features of intentional injury should be known and sought and vital clues can be found in the history, physical examination and common patterns of presentation.

Contemporary burn management is underscored by several decades of advancing medical and surgical care however, common to all injuries, it is in the area of prevention that the greatest potential to reduce the burden of these devastating occurrences exists.

## Introduction

Contemporary understanding of burn management in the pediatric population is underscored by several decades of advancing care in resuscitation, reconstruction and rehabilitation. Burns, however, remain a common and potentially devastating cause of injury in childhood.^1^

In addition to the treatment efforts of caregivers and Clinicians, primary and secondary preventative methods to reduce the incidence and severity of burn injuries have likely significantly impacted on the burden of injury evident in this population.

This review aims to discuss the epidemiology and risk factors associated with pediatric burn injuries and outline the mechanisms of injury sustained in patients, the assessment of the burned child, including disposition and psychological support, and finally the methods of prevention aiming to limit damage from this potentially debilitating injury in the pediatric population. Children may sustain burn injury through a variety of different energy sources which include thermal, electrical or chemical. These will be discussed in turn as well as the important case of intentional injury, an omnipresent consideration in presentations of pediatric injury.

**Epidemiology and Risk**

Burns are the third most frequent cause of childhood injury resulting in death, after motor vehicle accidents and drowning.^2,3^Whilst non-fatal burns vastly outnumber fatal burn injuries significant repercussions are possible from all burns for not only the child, but the family who may invest significant time and finances during long recovery periods.^4^Burns account for the greatest length of stay of all pediatric hospital admissions for injuries^2^and costs are substantial, with many hours of wound care and follow-up visits necessary, sometimes lasting months to years.^5^

The incidence of burns in various age groups has a bimodal distribution with children 0-4 years accounting for approximately half the number of burn accidents and the number rising again as adolescents sustain activity and work-related injuries.^5^Boys are also more likely to incur injury than girls.^1,6,13^The majority of these injuries occur in the home. Scalds resulting from hot liquids are most common (80%), occurring five times more frequently than those resulting from flame in the first three years of life.^7^Scalds also result in injuries of greater severity as reflected by nearly 25% of cases requiring hospital admission compared to less than 1% of other thermal burns requiring admission following initial treatment in one study.^13^Other causes of injury are contact with hot surfaces such as oven doors, fireplace screens, irons and hair care products.^1^In developed countries, scalds and contact burns are the most common mechanisms, whereas in developing countries cooking fires gain primacy.^8^

 Cases of child abuse are an important manifestation of burns owing to their very nature and not uncommon occurrence.These injuries account for approximately 6-20% of all abuse cases,^9^and severe burns are reported in an estimated 10% of all children suffering physical abuse.^10^

Exposure to electricity is a less common cause of burn injury, accounting for 2-10% of burn centre admissions, but these injuries may often result in amputation, and thus are a significant injury modality.^11,12^

In relation to the home environment, most burns occur in the kitchen involving food preparation and mealtimes^4,13^and seasonal variance indicates the winter months as a time of increased risk.^1,6^Injury may occur whilst under the supervision, of one or both parents.^4^Additional risk factors are low-socioeconomic status, low educational level of the primary care giver, home crowding (as estimated by number of household rooms) and psychosocial family stress.^6^

Hospital admission is required for only 6% of burns in children with most cases treated by primary care and emergency medicine physicians as reported by one study.^14^Factors that increase the severity of burns include aforementioned scalding injuries, younger age, increased burn size, and presence of inhalation injury.^15,16^

Inhalation injury is cited by some reporters as the single most important predictor of mortality in burn victims and occurs in 50% of children less than nine years old involved in home fires.^2^Another important predictor of death is deficiency or delay in resuscitation^16^which has been measured as length of time to intravenous access. Patients receiving resuscitation within the first hour having significantly higher chance of survival.^2^Children aged 0-4 are reported in some studies as having higher risk of death independent of burn size, possibly due to immature immune systems and increased fluid requirements placing young children at risk of sepsis and hypovolemia.^15^Respiratory failure and sepsis are the leading causes of death in severely burned children, with acute lung injury and respiratory distress syndrome (ARDS) accounting for 40-50% of all deaths.^16^Of particular concern is the finding that multi-drug resistant organisms increase death rates from patients with burn-related sepsis from 42% to 86%.^16^

**Mechanism of Injury**

Children are able to sustain burn injuries in a variety of ways in many common domestic settings. An understanding of these enables better assessment and ultimately prevention of burn presentations.

Injuries in pediatric patients are the result of behavior that can be intuitively related to developmental stages and epidemiology data. Toddlers, in which the highest overall rate of injury exists, are becoming mobile and are actively searching their physical environment. They are yet, however, to learn of the dangers inherent in their behavior and readily encounter hazards in the home. When motor skill development outpaces cognitive development, disaster may result.^3,13^These burns are often reported to the social services agencies because they reflect inadequate supervision of the child by the care provider. Burns in older children and teenagers, especially boys, are often associated with risk-taking behavior such as careless use of flammable substances and experimentation with fireworks.^18^Studies have shown that young adolescent males occupy the population most at risk from flammable liquids, usually petrol (85%), with the need for debridement and grafting in nearly half (46%) of cases and hypertrophic scarring in an even greater proportion (56%).^19^Rates of these occurrences are also higher in school age children with Attention Deficit Disorder.^20^

Skin damage may result from a variety of different mechanisms whereby temperatures in excess of approximately 49ºC causes cellular damage, though the exact temperatures required depends on contact time. The type of injury sustained can be thermal contact burns from hot objects or fluids (termed scalds), flame burns, chemical burns, electrical injuries, and friction burns. The latter is of increasing significance in the context of exposure to treadmill belts with which toddlers commonly come into contact with their hands or feet, resulting in deep burns to delicate joint surfaces.^14^

Thermal burns are most commonly the result of liquids and patterns of injury seen are described as spill, flow or immersion injuries.^10,21^Spills occur when hot liquids fall from a height on to the victim and therefore commonly effect the head, neck and upper torso.^13,22^These injuries have irregular margins and non-uniform depth with downward flow evident by deeper burns in the superior regions, becoming shallower as the fluid cools.^22^

Young children are mentally inquisitive and physically unstable and they sustain burn-related injuries having applied downward traction on cords or handles in the kitchen.^1^Common scenarios resulting in scalds are children reaching up and pulling receptacles containing hot liquids onto themselves, or overturning containers resulting in spill or flow injuries. Children colliding with other persons holding hot liquids results in burns in few occasions^13^as does exposure to hot bathing water.^23^Peak times for scalding occurrences have been reported as highest between 6am and 9am, with smaller peaks at 11am and 6-8pm, which correlates with times adults are commonly preparing hot drinks.^21^Instant noodle containers and contents, particularly in their original polystyrene containers, remain dangerously hot when ‘ready to eat’ and constitute a true classic source of burn injury in children with minimal carelessness or inquisitive behavior required for serious injuries to occur.^24^

Liquid immersion burns occur when the child falls or is placed into a container of hot liquid. As the patient struggles, splash marks, indistinct borders and variable depths of injury develop in accidental instances.^22^This is very common in countries such as Mexico where the kitchen is small and crowded and cooking activities often occur at ground-level. Immersions that are deliberate incur burns of uniform depth with distinct borders with skin-fold sparing amongst other important features.^22^

Contact with hot objects is the second most commonly occurring burn mechanism in small children,^5^and in order to sustain a burn either the temperature must be extremely high or the contact abnormally long.^23^Contact thermal burns may result from irons, ovens and fire grills^25^with upper limbs mostly effected.^13^Household appliances and devices posing well-documented hazards to children and potential opportunities for prevention include hot irons, woodstoves (particularly when uncovered) and oven doors.^26,27^The latter may be modified as ‘touch safe’ to reduce or eliminate the morbidity of these exposures. Children of all ages are at risk of contact burns from motorcycle exhausts, which, despite the usually modest burn size inflicted, possess great potential for cosmetic deformities and social implications in this age range.^28^

In many countries and climates, outdoor camping is a popular recreational pursuit with studies from Australia for instance reporting one million nights a year spent in one State’s national parks (Queensland).^17^Hot embers from campfires that may remain sufficiently hot to cause a full-thickness burn after one second exposure following attempted extinguishment using sand for up to 8 hours, which is often well into the next morning.^17^As the danger is disguised, this is a particular hazard for children. Water, in comparison, results in a rapid drop in temperature of the fire to 47 ºC (range 46ºC-50ºC) by ten minutes and is the only safe way to extinguish a campfire.^17^

Children posses the motor function to strike a match or lighter but lack the cognition to comprehend the danger involved. They are able to initiate house fires in this context and sustain flame burns associated with deeper burns and inhalation injury.^5,23^An additional risk in pediatric patients is that often they may not be able to escape from the burning object or room in these instances.^5^

In children up to two years of age the most frequent mechanism of electrical injury is direct oral contact through biting a cord. Less commonly, objects can be placed into power outlets, located within easy-reach at floor level.^25^After this age, outdoor risk-taking activities such as climbing trees and utility structures exposes older children to high-voltage electrical sources.^29^

Chemical burns can be sustained from contact with or consumption of corrosive agents typically stored within reach of the child under sinks or in cabinets, such as household cleaners, lye or bleach.^25^

**Thermal Injury**

Children less than 6 years old have thinner skin than do older children and adults and they are therefore at higher risk for burn injury even when exposure time is short.^1^As previously discussed, scalds are the commonest thermal injury sustained in childhood (80%) and it is estimated that it takes only a quarter of the time for a child to sustain a scald compared to an adult.^10^The average temperature of a hot beverage is 160ºF to 180ºF (71-82ºC) yet children exposed for 5 seconds to water at 140ºF (60ºC) will sustain a full-thickness burn.^30^Scald injuries can be caused by any type of hot liquid including tap water, tea and coffee and thicker liquids such as soups, grease and tar,^22^resulting in spill or immersion patterns.

Causal and physical characteristics define the features of a scald. Causal factors include the thermal agent, mechanism and intent of the injury. Physical appearance refers to the pattern with respect to depth (superficial, deep dermal, full thickness or mixed), the outline, the distribution referring to the affected body part and the extent according to the total body surface area affected.^10^

The classifications of burns commonly grade depth as first, second or third degree, and more descriptively as superficial, partial thickness or full thickness, respectively.^18^It is common to have several depths evident in these heterogeneous injuries.^18^

Superficial burns are classically erythematous and painful involving the intact dermis with no blistering. They heal without scarring and fluid loss is not a critical consideration.^18^Partial thickness burns are further divided into superficial or deep. The former involve partial destruction of the dermis, appear red with blistering, and are painful. These burns are moist and healing occurs in 7-10 days with minimal scarring. Deep partial-thickness burns penetrate to greater than 50% of the dermis. Nerve fibres are destroyed rendering them less painful and they have a white, pale appearance taking 2-3 weeks to heal.^18^Full-thickness burns appear white, waxy or leathery with no bleeding or demonstrable capillary refill. They are the most severe, are painless and place the patient at high risk of severe fluid loss and infection.^18^

When destruction of underlying structures such as tendons, nerves, muscles, bone and fascia occurs the burn may be referred to as fourth degree.^18^

The physiological functions of the skin are well known and greatly appreciated. Loss of the integrity of the skin, as occurs in burn injury, compromises the integument’s function to protect the body from infection, regulate body temperature and prevent body fluid loss via a barrier mechanism.^18^The sequelae that follow a burn injury are logically related to loss of these functions as well as systemic responses following cytokine release in burns greater than 30% total body surface area. These responses alter the cardiovascular system, manifest as increased tissue permeability and decreased myocardial contractility; the respiratory system seen as bronchoconstriction and ARDS; metabolic changes with an increase basal metabolic rate evident; and immunologic compromise.^23^

Local responses can be described using the 3 zones of a burn as described by Jackson in 1947. The first of these is the area of maximum damage termed the zone of coa-gulation where irreversible tissue loss occurs. The surround-ing tissue, the zone of stasis, is characterized by de-creased tissue perfusion where additional insults such as hypoperfusion or infection can cause complete tissue loss. Finally, the outer zone of hyperemia surrounds the injury where tissue perfusion is increased. This outermost zone will invariably recover.^23^

**Electrical Injury**

The most common etiologies of electrical burn injury in children differ from adults and usually occur at home accounting for 3-9% of all admissions to major burns centres in one study.^11^

Electrical burn injury is classified into one of two groups, low voltage (less than 1000V) and high voltage (more than 1000V) with the former associated with the home where electrical cords can be bitten, particularly by younger children. High voltage injuries are a serious problem in adolescent boys engaging in high risk behavior around power lines or from lightening strikes.^12^ Both forms of injury carry serious morbidity as measured by permanent defects, amputations or operative procedures,^13^with instances of death being from cardiac arrest following acute cardiac arrhythmias at the scene.

Electrical injuries differ from other types of burns in that major damage may be concealed and visible areas of necrosis represent only a small proportion of the tissue destroyed.^11^Fortunately younger children are rarely exposed to high voltage electricity and sustain burns from house current which are usually limited in depth and extent.^7^A wide range of physiological responses may be produced ranging from neural and muscular excitation with no tissue damage through to complex deep trauma involving multiple tissue types. Children have an overall lower fat component and different surface area to volume ratio which impacts on the risk of deep tissue damage compared to adults with equivalent injury.^12^

Thermal injuries seen following electrical exposures vary in proportion to the degree of electrical current. The extent of injury is dependent on factors such as resistance of tissues encountered, type of current, duration of contact and the pathway taken.^18^Resistance may be inversely proportional to tissue injury with electricity preferentially flowing through low-resistance structures such as nerves, muscles and blood vessels causing severe damage.^18^When exposed to high voltage, high resistance tissues such as tendons, bone and fat generate greater heat and predictions of the extent of these injuries is notoriously difficult.^29^

Other factors that determine the nature of the injury are type of current and path of flow through the body. Alternating current produces muscle tetany through continuous contraction and relaxation with each cycle and is thus more dangerous than direct current.^18^The alternating nature of domestic current can interfere with the cardiac cycle and give rise to arrhythmias,^23^as can high voltage exposures which can cause ventricular fibrillation or asystole.^29^These usually occur at the time of injury.^11^Flow through the body usually follows three potential pathways, hand-to-hand, hand-to-foot and foot-to-foot. Hand-to-hand is the most dangerous pathway due to risks of spinal cord transection at C4 to C8, myocardial damage and suffocation due to chest wall muscle tetany.^18^Flash injury is possible when an arc of current produces heat sufficient for superficial burns with no current passing actually through the body.^23^

Sequelae associated with electrical burns include muscle necrosis, associated trauma and perioral complications, more specific to low-voltage oral burns. Heat injury, diffuse vascular damage and intense swelling within fixed muscle compartments all lead to muscle necrosis.^11^Compartment syndrome associated with high-voltage injuries has been associated with amputation rates of 50%.^29^Associated fractures and visceral injuries from electrical burn injury is common following transient loss of consciousness or the strong, sudden contraction of muscles evident in high voltage exposures.^11^As mentioned, oral burns are common following the frequent incidence of children inserting a live electrical cord into the mouth. Contracture of the oral commissures may result, severely limiting the motion of the patient’s mouth.^32^these oral burns can extend entirely through the lip and oral mucosa and if the necrotic zone extends into the labial artery, commonly 10-14 days after the burn, result in severe bleeding.^14^Parents should be warned of this risk and instructed on methods of occluding the vessel.^5^

**Chemical Injury**

Exploratory tasting of household products is a common occurrence in the pediatric population however chemical burns only have a morbidity and mortality of collectively 1%.^18^Burns may result when a child swallows these often corrosive substances such as drain cleaners or other cleaning solutions. Injury is sustained to the mouth and esophagus and sometimes the upper gastrointestinal tract. Rather than thermal, the mechanism of tissue damage is usually a direct chemical reaction although the former may result from an exothermic reaction.^18,29^

Over 25,000 different chemicals can produce injury, the nature of which may be broadly classified as acid or alkali.^18^Acid burns produce a coagulative necrosis, which effectively limits the penetration of the offending substance and the depth of the burn. Common acidic household substances include drain cleaners (sulphuric and hydrochloric acid) and car batteries (sulphuric acid). Ingestion of products of increased acidity or in greater quantity will lead to gastric injuries and the potential for pyloric strictures.^18^In contrast, alkaline products result in liquefactive necrosis of tissues, which facilitates deeper penetration and causes a more significant burn. Products commonly encountered are lye (sodium hydroxide) and oven and drain cleaners (sodium and potassium hydroxide). Again significant gastrointestinal injury is possible, with the increased likelihood of perforation and more commonly, esophageal strictures.^18^

First aid is paramount and involves removal of contaminated clothing, copious irrigation regardless of the type of chemical burn to topical exposures and dilution in the form of a glass of water for chemical ingestions. No attempt should be made to induce emesis which re-exposes tissues, or to neutralize the original substance as this can provoke an exothermic reaction, worsening the initial insult.^14,18^These exposures are routinely dealt with by Poison Control or Information Centres and seldom present for medical assessment after appropriate first aid and referral advice is provided.

**Intentional Injury**

Burn injuries in which abuse is confirmed or suspected comprise a sizable portion of the pediatric burn population with incident rates from 10-12%,^10^though estimations vary between studies.^33^Between 10-20% of burn presentations are inflicted burns and the importance of awareness and recognition of these presentations is of paramount importance as the opportunity for intervention is vital considering 50% of children may experience recurrent abuse^9^and 30% of this group are eventually mortally injured.^23^Furthermore, reporting suspected abuse to the appropriate child protective authority is mandated by law for all physicians encountering these scenarios.^18^

Burn injuries that are intentionally inflicted are more extensive and severe^18^with longer hospital stays and increased intensive care admissions.^34^This is further reflected by a 30% mortality rate compared to 2% from accidental burns.^22^Children in these situations are, on average, between two and four years of age, more commonly are boys and come from low-socioeconomic households of two or more children, with most often the abused child being the youngest.^33-35^

A US multi-centre study conducted in 2008 identified a total of 2388 patients in the burn model system database less than 18 years of age. Patients were categorized into two groups: intentional injury (CPS) and non-intentional. The intentional burn injury patients were more likely to be African American, have a lower maternal education, with no paternal involvement, parental unemployment, and live with family members other than biological parents, and have higher rates of parental alcohol and drug abuse. In terms of physical characteristics the CPS group had injuries with higher proportions of trunk (78.9% vs. 68.6%, p was less than 0.001) and perineum (31.9% vs. 14.7%, p was less than 0.0001) burns, and surgery was required more often to the trunk (47% vs. 33.8%, p was less than 0.0001) and perineum (11.1% vs. 4.2%, p was less than 0.0001). Groups did not differ on number of operations, or prevalence of upper extremity burns.  The intentional injury patients were discharged more frequently to rehabilitation facilities (14.8% vs. 9.7%, p was less than 0.001).

The motivations and risk predictors for such horrific acts are interrelated, with acute family stress and lack of external support powerful precipitants of intense frustration and compounded in low-income, single parent families of multiple children. Abuse is symptomatic of dysfunction and the origins of such behavior in the abuser stems from poverty, desperation, substance dependence and potential previous abuse.^33^Frustrations often overcome the abusers emotional reserves, especially with small children who are at their most demanding and are also easy targets for maltreatment.^35^Frustrations are often vented as children can precipitate acute stress during periods of increased maintenance requirements such as toileting.

Abusive behavior is often an immature defense mechanism seen when needful persons react in an ill-considered way to resolve frustrations which can stem from environments devoid of emotional support, especially when needs for comfort, usually provided by spouse, partner or child are ungratified.^35^An understanding of the offender’s pain and sadness as a contributor to the eventual outcome of abusive behavior and subsequent injury can enable the healthcare provider to initiate more holistic treatment and critical, empathetic assessment.^35^

Clinical features often distinguish intentional from unintentional burns and assessment is grounded in a thorough history and physical examination together with observation of parental behavior and parental-child interaction. Features of presentation are important with 70% of inflicted tap water burn injuries having delay in care greater than 2 hours and a further 70% of victims being brought to care by a person other than the caregiver following injury occurrence.^36^Tap water itself is implicated in 83% of intentional scald injuries compared with 16% of general accidental injuries being caused by tap water alone.^22^The history must correlate with the child’s development, with age appropriate injury mechanisms verified against the child’s abilities.^34^Suspicion should be prompted by discrepancies or inconsistencies in the history as well as serial observation of parental behavior. Parents who are not abusive typically report details spontaneously with expressions of concern regarding prognosis and visit the child frequently, often bearing gifts. Offenders in contrast may be evasive, require prompting, appear critical of the child and lack remorse.^35^

Patterns of the majority of inflicted burns can be observed on physical examination(Table 1).These are immersion, splash, flexion and contact burns. Immersion injuries appear uniform in depth with sharp well demarcated lines that are often bilateral. Sparing of the buttocks as they are pushed against the floor of the tub results in the ‘doughnut’ appearance to the wound.^9^Simultaneous deep burns of the buttocks, perineum and both feet are pathognomonic of deliberate injury.^22^Splash burns occur when the scalding agent is thrown on to the victim. These are difficult to distinguish from accidental splash burns, as both are characterized by non-heterogeneous distribution of burn depth and unclear demarcation. This is a rare mode of assault however and non-intentional injuries tend to involve upper extremities, the face and the trunk with the ‘arrowhead’ pattern of decreasing severity as liquids cool with progressive skin exposure influenced by gravity.^9^Flexion patterns are seen when the victim’s joints are held in flexion from fear, pain or by others in forced immersion. These commonly involve the hips anteriorly and popliteal fossa posteriorly. The lower abdomen may also be spared when the trunk is in flexion when adopting a typical defensive position.^9,18^Partial sparing is also often seen around orbital and paranasal regions if the immersion injury involves the face. These patterns suggest that the immersions were brief in very hot water and exhalation of air from crying provided some brief protection.^36^Contact burns are characterized by the configuration of the burning object and in the case of intentional injuries are clearly delineated as the victim would otherwise move about in reaction to the insult.^9^Patterns seen can involve iron or cigarette burns and should raise concern for inflicted injury.^37^

**Table:** Table 1:****Prototype triage tool for diagnosis of intentional scalds: Reprinted from  Maguire, S., et al., A systematic review of the features that indicate intentional scalds in children. Burns, 2008. 34(8): p. 1072-81, with permission from Elsevier.

Intentional scald must be excluded	Intentional scald should be considered	Intentional scald unlikely
**Mechanism** – Immersion	**Pattern**– uniform scald depth; skin fold sparing; central sparing buttocks (doughnut)	**Mechanism** – spill injury; flowing water injury
**Agent** – Hot tap water	**Distribution** – glove and stocking; 1 limb glove/stocking	**Agent** – non-tap water (beverage)
**Pattern** – clear upper limits; scald symmetry (extremi-ties)	**Clinical Features** – previous burn injury; neglect/faltering growth; history inconsistent with assessed development	**Pattern** – irregular margin and depth; lack stocking distribution
**Distribution** – isolated buttock/perineum, +/- lower extremities; isolated lower extremities	**Historical/Social features** – trigger, such as soiling/enuresis/misbehavior; differing historical accounts; lack of parental concern; unrelated adult presenting child; child known to social services	**Distribution** – asymmetric involvement lower limbs; head, neck and trunk or face and upper body
** Clinical Features **– Associated unrelated injury; history incompatible with examination findings		
**Historical/Social features ** – Passive, introverted, fearful child; previous abuse; domestic violence; numerous prior accidental injuries; sibling blamed for injury		

A systematic review of features indicative of intentional burn injuries and features making abuse a less likely burn mechanism was completed by Maguire et al. The triage tool featured in Table 1 was suggested by these investigators and reflect the risks factors and protective factors for this aetiology.^10^

Acid-throwing constitutes a special case of intentional injury displaying an alarming increase in cases over the past 2 decades in Bangladesh. Readily available agents are used against females usually aged between 12 and 15, again in low socio-economic situations.^38^Attacks are perpetrated by men against women with 47 cases in 1996 increasing to 228 in 2001 commonly over disputes over rejected sexual advances or martial disputes (41%), land and family disputes (32%) and dowry dissatisfaction (13%).^33^Isolated cases of disputes between neighbors have culminated in acid attacks on babies.^33^

**Self-inflicted Injury**

Whilst an uncommon presentation, in pediatrics, burns can be self-inflicted in an attempt at suicide (self-immolation) or as part of a deliberate self-harm syndrome, consisting of continual, sudden urges towards self-harm. These acts have received increased attention in recent years with a consistently higher rate in women in studies performed in developing countries.^39^

This behavior is often associated often with intolerable situations involving a perceived loss of control and over-whelming the patient’s ability to cope.^39^Common associations are substance abuse, female sex, a lack of social support and eating disorders, which constitutes the most likely overlap with the younger population.^39^Self-immolation suicide is predominately a method chosen by men, the young and the severely mentally ill^39^as well as a psychopathologic family dynamic and a possible adherence to fundamentalist religious convictions.^39^

Common findings in the history of a patient with self-inflicted burns are previous psychiatric problems, often depression or borderline personality disorder, a failed suicide attempt or poor response by others to suicide ideation and a recent life stress.^33^

One of the highest rates of this behavior is evident in Iran amongst young females where marital conflict is a significant risk-factor. Others include low-literacy and socioeconomic status, limited access to mental health services and post-traumatic stress disorder.^34^

**Clinical Examination and Evaluation**

Evaluation of any burn presentation must first consider that these injuries may present as emergencies requiring immediate resuscitation and assessment prioritized to ensure care and survival of the patient whilst nonetheless undertaking a thorough assessment and evaluation.

**Burn surface area estimation and resuscitation**

Regardless of the etiology of the burn, the initial assessment should first include airway, breathing and circulation with mindfulness of the considerations of these parameters in paediatrics.^18^Routine signs of hypovolemia, such as decreased blood pressure and urine output are late manifestations in children and tachycardia is omnipresent.^2^The cardiopulmonary reserve in children is extraordinary and 25% of circulating volume may be lost before clinical signs appear and decompensation is imminent. Cool clammy extremities, mental obtundation and delayed capillary refill are signs of a child in significant danger.^2^

Fluid resuscitation must be commenced as soon as possible if indicated and the need for this must be anticipated, therefore intravenous access should be obtained immediately.^18^This may be difficult in the absence of intact integument, and intraosseous access is an acceptable resort in young children without acceptable venous sites.^18,29^At this time, laboratory data may be obtained, including full blood count, electrolytes with blood urea nitrogen and creatinine and liver function tests.^18^Children possess a diminished weight to body surface ratio and fluid losses are proportionally greater.^2^

In order to guide dosage calculation of fluid requirements, and for a detailed risk assessment, total body surface area (TBSA) of the burn must be estimated. Areas of superficial burning, as previously described, should not be included in the surface area calculation as they do not contribute to the risk of intravascular fluid loss.^18^An accurate estimation of the depth of skin injury often requires removal of devitalised tissue, which is further indicated to reduce the cytokine and inflammatory mediator load.^5^This debridement is often intensely painful and appropriate analgesia should be provided. Bear in mind that children do not always express pain in the same way as adults, sometimes displaying fear, anxiety, agitation, aggression or withdrawal.^2^Blood flow may be decreased to tissues and therefore subcutaneous or intramuscular administration should be avoided.^5^Only small, isolated blisters should be kept intact in this process as blister fluid contains mediators that promote dermal ischemia.^5^Children are proportionally different to adults, with a greater head size and relatively smaller limbs rendering the classic ‘rule of 9s’ for assessment of TBSA inaccurate.^18,29^Estimations in pediatrics can be undertaken using the Lund and Browder chart, or more simply by using the child’s palm, including fingers, as a representation of 1% TBSA in all ages.^18,29^

These qualitative methods are routinely becoming superseded as Laser Doppler Imaging technology gains acceptance as the preferred clinical predictor tool in TBSA prediction. LDI produces a color-coded image of skin blood perfusion using a low intensity red laser beam which penetrates the full dermis reflecting both moving red blood cells (RBC) and static tissue. Perfusion units are assigned to quantify RBC movement through tissue vessels which are translated into colors transposed onto a digital image of the burn.^39^This technique has been shown to correlate well with the gold standard of burn depth assessment, which utilizes histological markers on biopsied tissue. It has also demonstrated accuracy for predicting wound outcome in pediatrics and can be used accurately within the first 24 hours post-burn and following use of dressings and antimicrobials such as nanocrystalline silver dressings and silver sulphadiazine cream.^40^

System-wide extravasation of fluids into unburned tissues also occurs when burn size exceeds 20% total body surface area which, coupled with evaporated loss, leads to burn shock requiring intravascular fluid replacement in the form of isotonic crystalloids. The standard burn formula for children is derived from the Parkland formula developed for adults in the 1960s.^5^

Estimated Fluid Volume = 3 x Body Weight (kg) x %TBSA + Standard Pediatric Maintenance Fluids.

The use of colloidal replacement fluids should be avoided acutely unless serum albumin falls below 1.7g/dL as proteinaceous fluid leaks from capillaries and administering proteins may exacerbate the tissue edema by increasing extravascular osmotic pressure load.^5^

**Head-to-toe assessment**

A thorough Head-to-Toe assessment should be undertaken, assessing for associated injuries. This step is especially important if the history indicated electrical burn injury from a high voltage source as traumatic injury is commonly sustained in these instances^11^Electrical injuries may also affect multiple organ systems. Cutaneous manifestations include flame, flash and arc burns as well as mottled, cyanotic skin due to autonomic instability.^18^

Limbs and extremities, as well as the thorax, should be carefully assessed for circumferential burns. Careful observation of these injuries is imperative to detect the development of compartment syndrome secondary to muscle swelling within constricted facial compartments.^29^Often performed prophylactically, evidence of decreased perfusion is indication for immediate incision of the burned skin (escharotomy) to restore limb perfusion.^5,29^

**Additional monitoring**

Temperature monitoring is indicated following burns presentations as routine methods of heat conservation are inadequate following major compromise of integument.^2^Infants and toddlers are particularly susceptible due to increased surface area/volume ratios, decreased insulating fat and lower muscle mass used for shivering. Burned clothing that is hot should be immediately removed. A clean sheet should then be used to cover the burns during assessment, using caution to prevent the complication of hypothermia.^18^In this instance, the oxyhemoglobin curve is displaced leftward, impairing peripheral oxygenation to already compromised tissues. Furthermore, ventricular arrhythmias are not uncommon and myocardial susceptibility to electrolyte imbalances is increased. Burned patients often strive for temperatures of 38 ºC and depressed or even normal temperatures may be a sign of physiologic exhaustion and should be viewed as an ominous sign.^2^The burned wound is a potent source of pyrexic mediators and fevers are frequent in burned children. Every fever spike does not demand an extensive evaluation however, the wounds require close monitoring for evidence of cellulitis, increased drainage or discolouration.^5^The initial use of systemic antimicrobials is no longer indicated as trials have demonstrated they do not protect against subsequent infection and may contribute to resistant organism development.^5^

An indwelling catheter is essential for burns greater than 20% to allow fluid resuscitation to be titrated to achieve urine output of 1mL/kg/h. Catheterization is also often indicated in the instance of electrical injuries where significant muscle damage may give rise to rhabdomyolysis causing renal failure. High risk patients include those with suspected significant muscle damage or pigmented urine.^18,23^

A history consistent with electrical exposure necessitates cardiac assessment due to the possibility of arrhythmia. Changes usually occur concurrently with the injury and there is good evidence that if the patient has a normal EKG on admission and there is no history of loss of consciousness cardiac monitoring is not required. High voltage injuries, LOC or EKG abnormalities at admission require 24 hours of continuous cardiac monitoring.^11,18,23^Enzymatic indicators of myocardial damage should be assessed, remembering that CK and CK-MB elevations are largely of musculoskeletal origin.^11^

**Inhalation injury**

The airway should be evaluated immediately for potential compromise when smoke inhalation injury is suspected in any burned patient. Burns caused by flame or with a history of smoke exposure are high risk. Heat is extremely well dissipated in the upper airways and the pathophysiology of the injury is related not to thermal damage but severe inflammation due to particulate components of the smoke. ^5^Consequent edema may not be apparent until 48 hours after a burn and subsequent intubation is under the most difficult circumstances. Airway compromise should be anticipated and the airway secured via endotracheal intubation in the presence of stridor, hoarseness, carbonaceous sputum, singed nasal hair, eyebrows or eyelashes or perioral/nasal burns.^18^The urgency of airway management is further heightened in pediatric patients who possess a smaller aperture trachea which is predisposed to obstruction exhibiting disproportionate increases in resistance with equal amounts of edema compared to the adult.^2^

All patients who are victims of house or indoor fires should be evaluated for carbon monoxide toxicity, keeping in mind that the classic ‘cherry red’ appearance of carboxyhaemoglobin is often unapparent or obscured by the burn. 100% oxygen should be administered in this instance.^18,29^

**Features suspicious of intentional injury**

The assessment should always include an open mind for the possibility of intentional injury, which should be thoroughly investigated when clinical features, as previously detailed, present in the history and examination. The history of the burned child is of particular importance in assessing whether the burn is likely to be accidental or more sinister. Reported mechanism of injury must be carefully correlated with the observed pattern of injury, burn depth and appearance on physical examination.^22^The presence of witnesses and the exact timing must be confirmed and these persons interviewed to assess for correlation in narratives.^22^

Abused children share characteristics of age (being in-fants or pre-schoolers), inconsolable crying, toilet training or associated toilet accidents, parent-child bonding impairment, inappropriate behavior such as apathy or apparent tolerance to invasive procedures and poor record of immunisations.^9^Similarly, abusers also possess many common traits being commonly adolescent parents, often maintaining inconsistent expectations for child’s development, experiencing a lack of external supports, stressors such as substance abuse, unemployment, poor housing and mental illness, being reliant on children for emotional support, single and possibly abused themselves.^9^A thorough event reconstruction soon after admission should be conducted and documented. This limits the room the suspect has to alter the events and timeline related to the burn or the opportunity to collude with witnesses.^9^The pattern of circumstances is extremely important with suspicions raised when the adult responsible claims not to have seen the incident, attributes the injury to a sibling, presents late or relatives other than the adult supervising at the time of the burn bring the child for assessment.^33^A health care professional that has established sufficient rapport should, at an appropriate stage, interview the child alone. Children even as young as 29 months may relate information with remarkable accuracy.^35^

Aspects of the physical exam have been previously mentioned with pattern, distribution and associated features being the more important features to determine. No published studies have validated the practice of dating a scald and therefore the comment that a scald is older than the history stated cannot be substantiated, though experienced clinicians may feel confident in doing so.^10^

If abuse is suspected in a child less than two a skeletal survey must additionally be conducted to assess for occult fractures.^37,41^Despite suggestions patients with burn injuries may constitute a lower risk group for associated fractures from abuse, 18.6% of children in this subgroup were found to have fractures at skeletal survey in one study.^41^There is evidence that children with burns are less likely to be evaluated for fractures reflecting a perception that they represent lower risk, however the numbers are sufficient to support the recommendation for routine investigation for children less than two years old.^41^The clinician must also be mindful at this time and assess for signs of abusive head trauma such as intracranial and retinal haemorrhage.^37^

**Documentation**

It should be remembered when examining a burn the real possibility that litigation may follow, whether civil or criminal, as well as the obligation to report cases of suspected abuse and neglect and the possible investigation of acts or omissions in this regard. Clear and careful notes are therefore necessary and all aspects of the physical examination should likewise be carefully recorded and, if possible, photographed to allow for future review.^33,37^Treatment and time may change the appearance of burns, so photographic evidence of appearance at presentation is an important consideration if the situation allows.

**Disposition**

Several criteria have been suggested for recommending transfer of children to a multidisciplinary burns specialized burns centre. A burn covering greater than 10% TBSA in a child under 10 years is a critical burn and should be transferred to a dedicated burns unit. In addition, burns affecting the face, hands, genitals and perineum, feet or across joints and co-existing inhalation injury, trauma or child abuse should be referred.^5^Other instances requiring consultation with a burns unit are electrical burns, circumferential burns and cases requiring long-term emotional or rehabilitation support^18^Psychological assessment, support and treatment are mandatory aspects of multidisciplinary care provided in the pediatric burn population.^18^

**Psychological responses to burn injuries**

Experiencing a burn injury and enduring the painful treatment is often traumatizing to children and adolescents.^41^The psychological response to the trauma of the injury and subsequent treatment becomes more complex when the injury is intentional. Trauma symptoms include cognitive difficulties (forgetfulness, inability to concentrate), physical difficulties (pain, trembling, restlessness, muscle tension, rapid heart rate, light headedness or dizziness, perspiration, cold hands/feet, shortness of breath), behavioral difficulties (temper tantrums, irritability, helplessness, detachment, difficulty separating from parents, difficulty falling asleep and nightmares),  and emotional difficulties (feelings of fear or dread, worrying excessively, anxiety).^42^As a result of being exposed to the trauma of abuse and the burn event, children may avoid any or all stimuli that remind them of the burn event. They may demonstrate absence of emotional responses and appear to be dazed and unaware of present surroundings. These symptoms may develop into psychiatric mood disorders. Among these anxiety disorders the most common experienced following an intentional burn injury are Acute Stress Disorder (ASD) and subsequently Post Traumatic Stress Disorder (PTSD). Children may also experience significant depression and a profound change in the way they see themselves and the way they see the world.^42^

**Prevention**

The recognition that the majority of pediatric burns occur at home provides opportunities to significantly impact on the burden of these devastating injuries through prevention, which remains the single best management strategy.^2^Much of the progress towards reductions in burn-related deaths in recent decades can be attributed to improvements in medical and surgical care, however a more important component are interventions that alter the physical or social environment affecting incidence and severity of injuries encountered.^34^Adequate supervision of children remains a major prevention tool for avoiding accidents of all types.

It is clear that efforts towards burn prevention must be ongoing and innovative as, although the kitchen is perceived as dangerous for young children, unanticipated injuries still most often occur in this environment and the presence of a supervising person is not a satisfactory deterrent as evidence by only 18% of children being alone at time of accident in one study.^3,13^

Methods employed to impact on injury occurrence may be described as active or passive. Active methods involve the initiation of an endeavour such as education or awareness campaigns. These however require the consistent participation of the caregiver or individual and do not seem to be effective alone.^8,31^Passive interventions such as consumer product safety legislation are noted in studies to be more effective and do not require engagement of the target population.^8,31^Specific examples of this are lowering of water temperature settings and cookware safeguards. These, as well as better building codes resulting in safe electrical wiring, smoke detector requirements and sprinkler system installations have likely influenced burn injury severity in developed countries.^34^

Certainly the issue of water temperature is important in preventing scald injuries in children. Water heaters should be set to 120ºF (49 ºC) maximum which reduces contact time for a full-thickness burn from 30 seconds at 131 ºF (55 ºC) to ten minutes.^29^Anti-scald devices in faucets are also available and some jurisdictions have introduced legislation to incorporate such initiatives into all new homes.^49^

General advice to prevent scalds follows the understanding of risk profile and mechanism of injury and involves advising parents to exclude children from kitchens during food preparation or placing them in a secure chair or space, avoiding hanging electric cords, turning pot handles away from bench edges whilst using rear burners and avoiding the use of tablecloths and placements which can easily be grasped and pulled.^1,3^Other recommendations that rely on parental vigilance are continual supervision at bath times and never leaving hot liquids unattended.^3,18^

Functioning smoke detectors are an important fire safety initiative which can prevent house fires and reduce the risk of death in fire by 60%.^25,29^In 2/3 of home fires in which a child was injured or killed, no working smoke alarm was found.^50^Alarms not only must be regularly assessed for functionality but children need to be taught how to respond and practicing fire escape drills is an important family activity.^50^Children should be instructed that in case of fire, hot doors should not be opened and they should cover their mouth and stay low to avoid smoke inhalation.^29^

Methods to reduce the impact of electrical burn injury should initially target the most important injury mechanism, which is electrical cord tampering. Manufacturing safety standards for these items are varied and often non-existent so parents require diligence to keep children away and cover electrical outlets with plastic covers.^18^Chemical burns on the other hand can be prevented through correct storage of caustic substances out of reach of children. Cabinets should be able to be locked and products kept in their original containers.^18^

United efforts aiming to positively impact on a greater number of potential victims are logical strategies to foster burn riskmitigation behavior. It has been suggested that prevention programs for burns in young children can be rendered effective and sustainable by using local injury data to develop targeted community based prevention as evident in the Norwegian Harstad injury prevention study.^8^A systematic review of published accounts of such activities revealed a limited number of studies allowing conclusions to be drawn and a pressing need to evaluate such programs to reduce burns in children.^51^A useful framework for widespread application of injury counter-measures has been described by Cohen and Swift and, as applied to burns prevention, is described in table 2.

**Table:** Table 2: **Developed from Spectrum of Prevention – Cohen and Swift^13^**

1 -strengthen individual knowledge and skill	have clinicians advise parents about potential for kitchen scalds at 9 months - 2 years of age
2 –promote community education	Have communities institute a burns/scalds awareness day
3 -educate providers to improve their understanding of prevention	Require child care providers to have some injury prevention training that addresses all sources of injury
4 –foster coalitions and networks	Develop community coalition to build partnership approach
5 –change organizational practices	Encourage media to offer fixed timeslots for regular announcement of incidents
6 –influence policy and legislation	Require manufacturers to seek design innovations to eliminate/reduce hazards and to include warnings and instructions about the use of items around children

## Conclusion

Burns remain a significant cause of injury in the pediatric population worldwide despite increasing advancement in treatment of affected patients, as reflected by reductions in morbidity and mortality associated with these injuries.

Mechanisms of injury are often unique to childhood and adolescence and are related to an imbalance of motor and cognitive function related to ability to explore the external environment and appreciation of the potential dangers of this curiosity.

Appropriate assessment and evaluation grounded in contemporary knowledge and care must be performed for all presentations of burns in order to best medically manage these injuries. Assessment should be accompanied by an understanding and recognition of features of intentional injury in order that this all-too common presentation of abuse is suspected, investigated and managed appropriately.

Ideally, the best reduction in the healthcare burden of this devastating mode of childhood injury is achieved through preventative measures. Appreciation of the significance and mechanisms of burn injuries, it is hoped, will further encourage the development of prevention initiatives reducing the incidence and severity of childhood burns in future years.
